# The Influence of Shot Peening and Artificially Ageing Aluminium Alloy 7075 on Corrosion Behaviour

**DOI:** 10.3390/ma15093094

**Published:** 2022-04-25

**Authors:** Sebastjan Žagar, Primož Mrvar, Janez Grum, Roman Šturm

**Affiliations:** 1 Faculty of Mechanical Engineering, University of Ljubljana, Aškerčeva 6, 1000 Ljubljana, Slovenia; sebastjan.zagar@fs.uni-lj.si (S.Ž.); janez.grum@fs.uni-lj.si (J.G.); 2 Faculty of Natural Sciences and Engineering, University of Ljubljana, Aškerčeva 12, 1000 Ljubljana, Slovenia; primoz.mrvar@ntf.uni-lj.si

**Keywords:** aluminium alloy 7075, shot peening, ageing temperature, pitting corrosion, cyclic polarisation, intermetallic particles

## Abstract

This paper investigates the corrosion of shot peened AA7075 aluminium alloys aged at different temperatures. The surface integrity of the hardened layer was evaluated with SEM, EDS, differential scanning calorimetry, hardness, and roughness measurements, and in the end also with corrosion resistance tests. The research results indicated that there were significant differences in precipitates distribution between aluminium alloys artificially aged at different temperatures. As the ageing temperature increases, the microhardness decreases, which influences the final roughness condition of the surface layer after shot peening. The results of potentiodynamic polarisation tests indicate that shot peening enables shifting the pitting potential to positive values, which ensures slightly higher corrosion resistance. SEM images confirmed the dissolution of the aluminium matrix near the separated iron-rich phases of the Al_x_ form (Fe, Mn) by the action of galvanic cells.

## 1. Introduction

Shot peening is a mechanical surface treatment process in which a thin layer of the workpiece surface is mechanically hardened. This surface treatment results in a characteristic surface topography and an increase of dislocation density and compressive residual stresses in the surface layer, which serve to inhibit or slow down the initiation of surface cracks and the growth of fatigue cracks [1,2,3]. Shot peening is a surface treatment commonly used to increase the efficiency of structural components made of high-strength steel [4], aluminium [5], and titanium alloys [6]. It improves fatigue and corrosion resistance and increases resistance to stress corrosion cracking [2,3,7]. The purpose of shot peening of surfaces is to extend the life of various machine parts. Important effects on the surface are closely related to the type of medium used, the available kinetic energy of the accelerated particles, the size and hardness of the particles, and finally, the degree of coverage of individual prints [8,9,10]. These particles are accelerated against the workpiece by means of various impact devices so that they interact with the workpiece surface [11]. 

The aluminium alloy AA7075 is a high-strength alloy with the addition of 3–7.5 wt.% zinc as the main alloying element in combination with magnesium and copper. These alloys have good formability and fairly good corrosion resistance [12]. The highest strength among all aluminium alloys, which even surpasses some steels, is achieved by artificial ageing [13]. These alloys are widely used for machine parts and load-bearing structures in the aerospace, automotive, and spacecraft industries due to their good weight to strength ratio and relatively good corrosion resistance [14]. However, the dynamic strength of these alloys decreases because they are very susceptible to corrosion fatigue, especially in saline environments [15].

Intermetallic phases formed by solution heat treatment are based on important alloying elements, such as zinc, magnesium, and copper. When the separate phases are concentrated at the boundaries of the crystal grains, they cause intergranular corrosion and increase sensitivity to crack formation and growth due to stress corrosion. Because of the presence of these alloying elements, it is assumed that the separated phases are close in chemical composition to the intermetallic compounds Al_2_Cu, MgZn_2_, Al_2_Mg_3_Zn_3_, Mg_2_Si, Al_2_CuMg, Al_7_Cu_2_Fe, and Al_13_Fe_4_ [16,17].

The susceptibility of these alloys to stress corrosion cracking as a result of thermal exposure is due to the migration of magnesium to the crystal grain boundaries and the possible formation of the magnesium-enriched β-phase of Al_3_Mg_2_, which is the anode with respect to the aluminium matrix [18,19,20]. 

High-strength aluminium alloys are susceptible to corrosion fatigue, especially in marine environments, resulting in a significant reduction in the fatigue strength [21]. Corrosion also has a significant influence on the ageing of structural elements made of high-strength aluminium alloys. Aluminium alloy 7075 in the Al–Zn–Mg–Cu alloy system contains a high number of intermetallic particles, i.e., constituent particles, for which heterogeneity of a microstructure has an essential influence on corrosion properties. Corrosion performance of aluminium alloys subjected to surface mechanical treatments has been extensively studied [22,23]. Pandey et al. [24] investigated the effect of the surface nanostructure developed through ultrasonic shot peening on corrosion behavior of aluminium alloy 7075. They found that the enhancement in corrosion resistance of an ultrasonic shot peened specimen is due to rapid development of a uniform, homogeneous, and effective passive layer on the nanostructured surface coupled with refinement of the coarse precipitates. Several researchers did mechanical surface treatment with ultrasonic shot peening. Safyari and Moshtaghi [25] researched the effect of ultrasonic shot peening on the environmental hydrogen embrittlement behavior of the 7075-T6 aluminum alloy. They found out that this treatment decreases the grain size in the surface layer. As a result, there is an increase in the grain boundary area and consequently a decrease in the number of atomic hydrogen trapped per unit length of the grain boundary. Sun et al. [26] studied microstructure, corrosion behavior, and thermal stability of ultrasonic shot peened AA7150. They concluded that for the studied environments, the corrosion resistance of peened AA7150 significantly improved compared with its untreated counterpart. Enhanced localised corrosion resistance is a consequence of the formation of equiaxed nano-grains and microstructure homogenisation on the surface peened layer. 

Ye et al. [27] analysed the influence of combined shot peening and plasma electrolytic oxidation treatment on the corrosion fatigue behavior of aluminium alloy 7A85. They concluded that with shot peening as a pre-treatment before plasma electrolytic oxidation treatment, the corrosion fatigue resistance of a multiple treated specimen was improved due to introduction of residual compressive stress, which can inhibit intergranular corrosion. 

Our research aims to investigate the influence of shot peening treatment on a chosen aluminium alloy subjected to different ageing temperatures by means of corrosion behavior. The research also addresses the influence of the precipitated phases on the corrosion resistance of the alloy. 

## 2. Experimental Procedure

### 2.1. Material and Specimen Preparation

The material selected for the analysis was a wrought aluminium alloy, AA7075 (AlZn5.5MgCu), which was supplied as a rolled plate with the dimensions 1500 mm × 1000 mm × 10 mm The chemical composition of the aluminium alloy is given in Table 1.

For metallographic specimen preparation, the specimens were cut to the dimensions of 40 mm × 40 mm × 10 mm on a milling machine. The specimens obtained were first solution heat treated at a temperature of 475 °C for 2 h. Then followed the process of quenching. After quenching, the specimens were artificially aged at three different temperatures (145 °C, 170 °C, and 195 °C) with a uniform ageing time of 8 h. The purpose of ageing at these temperatures was to obtain different densities and sizes of the individual separate phases. The ageing treatment was completed by taking specimens from the furnace and cooling them to the ambient air temperature. Tested specimens were labelled as follows: solution heat treated and quenched (QS), solution heat treated, quenched, and artificially aged at temperature 145 °C (AA145), solution heat treated, quenched, and artificially aged at temperature 170 °C (AA170), solution heat treated, quenched, and artificially aged at temperature 195 °C (AA195).

### 2.2. Shot Peening Treatment (SP)

A shot peened surface ensures the extension of the life of the machine part. Therefore, it is good to know the influence of individual hardening parameters. We measured surface roughness ten times, in various directions according to the rolling direction. The mean arithmetic value of the specimen roughness (R_a_) before shot peening was 0.28 μm. Shot peening was carried out with steel balls designated as S170 with diameters between 350–420 μm. The hardness of the media used was between 420 and 448 HV_1_. The specimens were clamped in rotating jaws through which high-pressure nozzles moved in an air stream with accelerated ejection of the steel balls. Shot peening was performed at an Almen intensity of 4A with coverage set to 100%. After shot peening, the roughness on all treated specimens increased, as well as the microhardness (Table 2). The thickness of the SP deformation layer is about 0.25 mm.

### 2.3. Corrosion Tests and X-ray Diffraction Analysis of the Surface

Potentiodynamic tests were performed in the range of −100 mV_SCE_ below the stable potential of the open circuit at a flying speed of 1 mVs^−1^ in the anode direction. The change of direction from the anode to the cathode was performed at a certain limit value of 1 mA/cm^2^. Typical potential values were then determined from the polarisation curve. To evaluate the cyclic polarisation, a VoltaLab potentiostat PGZ100 (Radiometer Analytical SAS, Villeurbanne CEDEX, France) and a CNC corrosion cell with a saturated calomel electrode (SCE = +246 mV) as the reference electrode were used. The electrochemical tests were performed in 0.15M (3.5%) NaCl solution with deionised water at pH (6.7 ± 0.1) at room temperature (22 ± 0.5 °C). The tests were performed according to ASTM G5-14 standard [28]. 

The diffraction spectra were recorded with a PANalytical X’Pert PRO (Malvern Panalytical Ltd., Malvern, UK) instrument in an angular range of 2θ from 15 to 90° using X-rays of the wavelength 0.154059 nm (Cu target) without the use of a monochromator.

## 3. Results and Discussion

**Figure 1:**
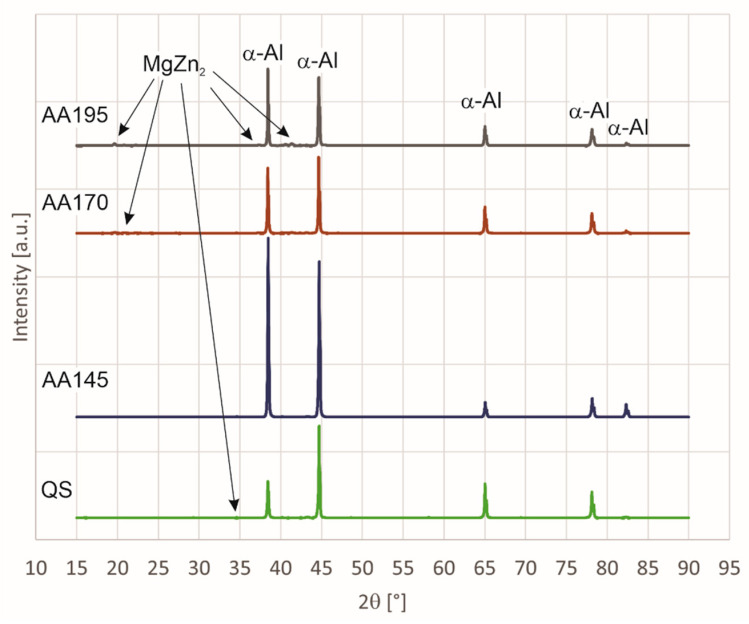
XRD diffraction patterns of AA7075 aluminium alloy subjected to various heat treatment conditions.

Figure 1 shows the diffraction spectra (XRD spectra) of all four 7075 alloy specimens (Al-Zn-Mg-Cu) tested, i.e., specimen in quenched state (QS), specimen artificially aged at 145 °C (AA145), specimen artificially aged at 170 °C (AA170), and specimen artificially aged at 195 °C (AA195). Among all specimens, the spectra of quenched state and of aged state at 195° C differ the most. In the spectrum, the first sharp diffraction peaks at 2θ angles of 38.5°, 44.8°, 65.1°, 78.2°, and 82.4° are from α-Al. The second less distinct peaks detected in the specimens are deduced as the MgZn_2_ (M-phase) [29], and these are at the 2θ angles of 19.5°, 20.7°, 34.3°, 37.3°, 39.8°, 40.5°, 41.3°, 42.3°, 46.9°, 53.5°, and 70.4°. The peaks at around 20 degrees do not appear on all curves. The peaks appear only on specimens AA170 and AA195 because the quantitative proportion of the MgZn_2_ phase is higher due to higher aging temperature.

The second peaks are most visible in the specimen aged at 195° C. Due to their small proportion and the overlapping of some peaks, the presence of the phases Al_2_CuMg (S-phase), Al_6_Mg_11_Zn_11_ (T-phase), Al_7_Cu_2_Fe, which are determined as insoluble impurity-originated particles [30,31,32], and Al_6_Fe should be further verified by electron microscopy and microanalysis. 

**Figure 2:**
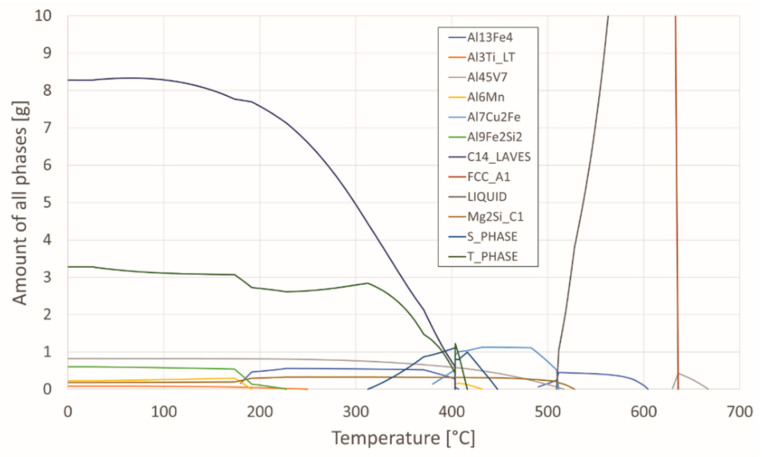
Proportion of phases as a function of temperature.

Phase equilibria was calculated from the chemical composition using the program Thermo-Calc and is shown in Table 1. Figure 2 shows the proportion of phases as a function of temperature for the investigated aluminium alloy. This can help interpret microstructures as a function of thermal treatment.

**Figure 3:**
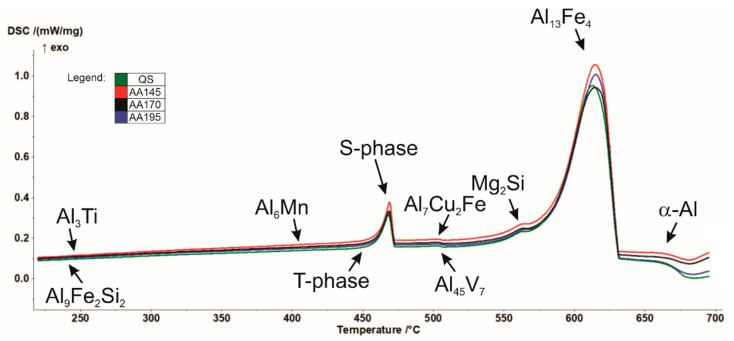
Cooling curves of the tested AA7075 specimens.

In addition, differential scanning calorimetry (DSC) was performed by means of spectral thermal analysis (STA). In Figure 3 cooling curves of the investigated specimens in the quenched and aged state (145 °C, 170° C, and 195 °C) are shown, attributed with phases calculated by Thermo-Calc. As can be seen from the thermodynamic calculation in conjunction with the results of the DSC analysis (Figure 2), solidification of the examined AA7075 alloy begins with the growth of primary α-Al mixed crystals. According to the calculation, this is followed by crystallisation of the remaining melt into the intermetallic phase Al_13_Fe_4_ in the temperature range from 604.4 °C to 490 °C. The phase C14_LAVES (designation according to Thermo-Calc) crystallises last at a temperature of 403.5 °C. This is the η-MgZn_2_ phase. Crystallisation of the T-phase starts at 447 °C, and the S-phase at 463 °C. We can find the similar description of the phases calculated by Thermo-Calc in the research [33,34].

In aluminium alloy 7075, precipitation proceeds as follows: Super Saturated Solid Solution (SSSS) → GP-zones (GPZs) → metastable η’ phase → stable η phase (MgZn_2_). At peak aged condition, the alloy contains the main precipitates of the metastable η’ phase transformed from GPZs. In the overaged condition, the main precipitates are the stable η phase [35,36]. 

**Figure 4:**
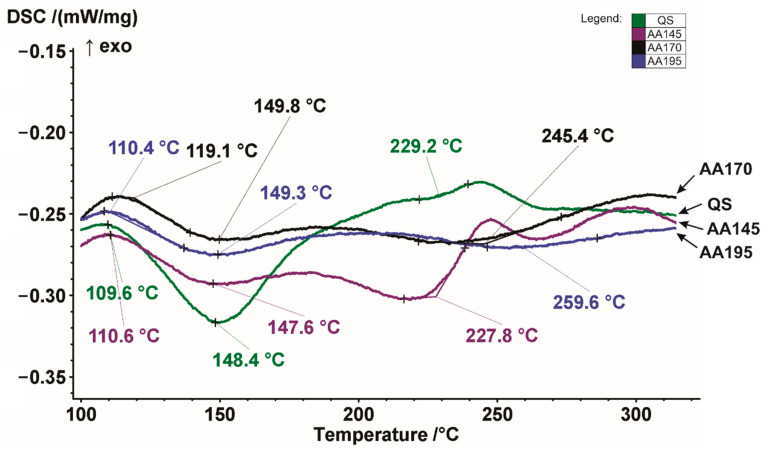
Detailed DSC heating curves of the examined AA7075 alloy in the quenched, and aged condition between 100 °C and 300 °C.

Figure 4 shows DSC heating curves of the examined AA7075 alloy in different states. 

The reference temperatures for the onset of decomposition of the precipitation hardened phases start at almost the same temperatures. The exception is the AA170 specimen, where decomposition begins at a slightly higher temperature. In the process, the intermetallic compounds formed during the ageing process decomposed in the order of precipitation hardening. Table 3 summarises exothermic precipitation and endothermic dissolution peaks for all selected heat treatment procedures. According to the research of Gjønnes and Simensen [37], the exothermic peaks are related to the precipitation phenomena, while the endothermic peaks are associated with the dissolution of precipitate.

From the quenched curve (QS) we can see that it has exothermic precipitation peaks and also endothermic dissolution peaks. It can be observed that the first exothermic precipitation peak is at a temperature 109.6 °C, and the first endothermic dissolution peak is at a temperature of 148.4 °C. The second less pronounced precipitation peak is at 220 °C, and the third exothermic precipitation peak is at 243 °C. The main difference between the quenched specimen and the artificially aged specimens appears in the first endothermic dissolution peaks, which are no longer so pronounced. The specimens aged at 170 °C and 195 °C show a similar trend, while the specimen aged at 145 °C deviates slightly from the previously mentioned specimens.

The endothermic dissolution peak appears at a temperature of about 220 °C, exactly where the exothermic precipitation peak appears in a quenched specimen. The exothermic precipitation peaks of quenched and aged at 145 °C specimens are then almost matched at 243 °C and 247 °C, respectively. The aged specimen has another exothermic precipitation peak at the end at 296 °C. The peak temperature in exothermic curve is an indication of the relative stability of pre-existing precipitates. Therefore, the precipitates responsible for the 1st exothermic peak continually form and grow and become more stable during room temperature ageing and artificial ageing. It also can be seen that the 1st exothermic peak increases with an increase in the ageing temperature. This reflects the rapid growth of zones during artificial ageing because the dissolution temperature is a function of the size of the precipitates. The sizes of the 1st and 2nd endothermic peaks decrease with increasing the artificial ageing temperature, and the 2nd endothermic peak has almost disappeared after ageing at 195 °C. This indicates that the zones are transforming to a more stable phase or dissolving as a more stable phase forms after artificial ageing. We can see that only when ageing at a temperature of 145 °C does a distinctly steep 3rd exothermic peak appear. This indicates that η’ is forming during the artificial ageing. When comparing the quenched state and ageing at 145 °C, we see that the position of the 3rd exothermic peak hardly changes, which indicates that the number density of the η’ precipitates increase during artificial ageing. Similar descriptions of exothermic precipitation peaks and endothermic dissolution peaks are reported in the research [38,39,40,41]. 

From the curves obtained, it can be concluded that the selected ageing temperatures chosen were too high, since the aluminium alloy shows signs of overageing and can therefore be assigned to the T7 state. Similar conclusions were taken by Takata et al. in his research [42].

**Figure 5:**
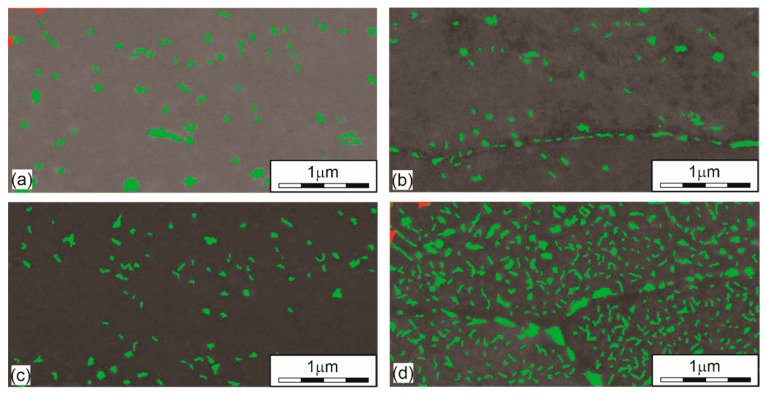
The amount of precipitated phases under different heat treatment conditions. (**a**) QS (3.63%); (**b**) T145 (2.93%); (**c**) T170 (3.05%); (**d**) T195 (15.16%).

Figure 5 shows precipitates in the microstructure of the aluminium alloy obtained at different ageing temperatures. Most precipitates are found at the highest ageing temperature of 195 °C (Figure 5d), occupying as much as 15% of the total surface. Larger precipitates are generated along the boundaries of the crystal grains. The quantity of precipitates in specimens in (a) a quenched state, (b) aged at 145 °C, (c) aged at 170 °C, and (d) aged at 195 °C has the values of 3.63%, 2.93%, 3.05%, and 15.16%, respectively. We can see that there are already quite large precipitates in the quenched state and that they exceed the percentage of precipitates in specimens aged with lower ageing temperatures, i.e., 145 °C and 170 °C. Figure 5a shows that the average size of the precipitates in the quenched state is around 100 nm and that not all of them are dissolved. So, we may conclude that the time of solution heat treatment was too short. With an aging temperature of 145 °C, the amount of precipitates decreases slightly while the average size of precipitates hardly changes (Figure 5b). As the aging temperature rises to 170 °C, as shown in Figure 5c, the amount of precipitates increases negligibly while the average size decreases to about 60–70 nm. These differences are related to the place of sampling. In the case of the highest aging temperature of 195 °C, we can see from Figure 5d that the size of the precipitates is from 30 nm up to about 300 nm. Larger precipitates, which are coarsed, are excreted just beyond the crystal grain boundaries, which also coincides with the study [43]. The main increase at this aging temperature is in the amount of precipitates, as it increases to a level of 15%.

**Figure 6:**
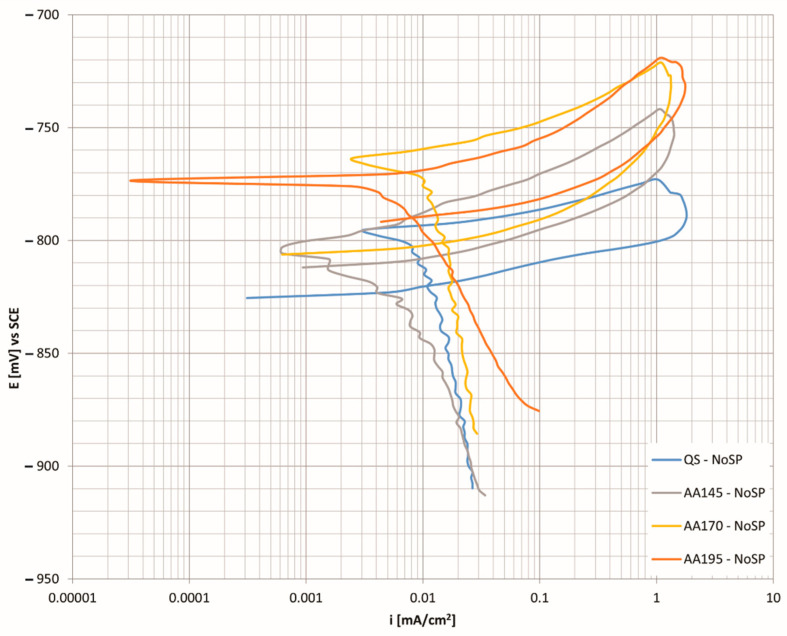
CP curves as a function of the corrosion current density for the unhardened state at different age hardening temperatures.

Figure 6 shows the cyclic polarisation curves for the aluminium alloy in different heat treatment conditions. The specimen aged at 145 °C proving to be the most corrosion resistant when compared to the other precipitation hardened states.

In addition to the low corrosion current, the curve is shifted to more positive values of the corrosion potential, which further contributes to corrosion protection. The specimens at higher temperatures come closest to precipitation solidification. In all test cases, a very similar trend of the cyclic polarisation curves was found. Such curve shapes are characteristic of pitting corrosion, which intersects the cathode area with a back fly-over. It is assumed that the tighter the hysteresis loop and the higher the value of the repassivation potential, the greater the resistance of the material to pitting corrosion formation. In general, it can be said that the lower initial surface roughness provides greater corrosion resistance to the material and that higher residual compressive stresses in the material inhibit the formation and spread of corrosion ulcers. Alloys of the 7xxx group are characterised by intergranular corrosion, which takes place at the crystal boundaries where we have precipitates associated with elements of copper or zinc, thus forming an area depleted of these elements. Among the factors influencing the corrosion resistance of aluminium alloys is the presence of chemical element Zn, which forms precipitates η (MgZn_2_), and is the most critical one [44,45].

**Figure 7:**
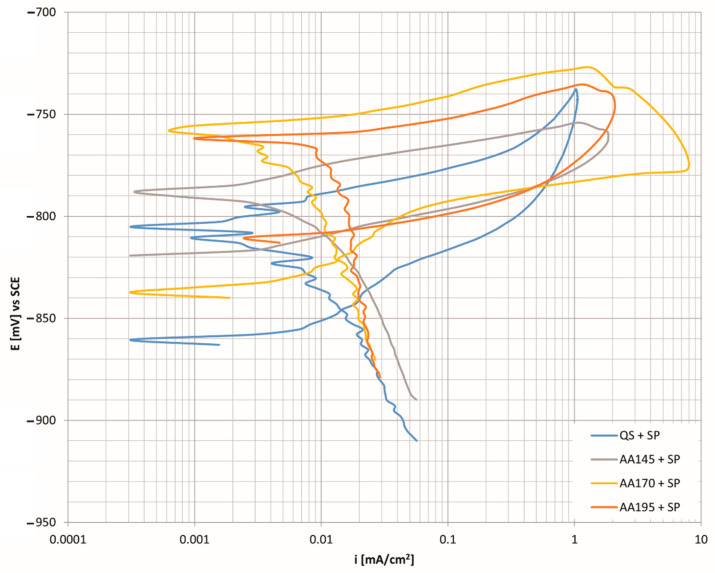
CP curves as a function of corrosion current density for Almen intensity 4A and 100% coverage at different age hardening temperatures.

Figure 7 shows the cyclic polarisation curves for an aluminium alloy shot peened with an Almen intensity of 4A and coverage set to 100%. The comparison of the corrosion current shows that the lowest currents were from a specimen aged at a temperature of 170 °C (i_corr_ = 0.0079 μAcm^−2^) and a specimen which was in the quenched state (i_corr_ = 0.0077 μAcm^−2^). The lower the corrosion current value, the greater the corrosion resistance of the material. Since the corrosion rate calculated per year is also directly related to the corrosion current, we also have the lowest values of material decomposition in these two specimens. The comparison with the age hardened specimen at a temperature of 145 °C shows that the latter has higher corrosion current and thus greater degradation of the material.

The analysis of the results of the electrical potential ΔE_1_ shows that the specimens in the quenched condition and aged at the lowest selected temperature have the greatest capacity for repassivation regarding the reprotection of corrosion pits during the previous anode overflight. The difference (Δ) between the hardened and unhardened alloy is 16 mV in the case of a quenched specimen and 14 mV in the case of aging at a temperature of 145 °C. At higher aging temperatures, the values are slightly higher, 27 mV at the ageing temperature of 170 °C and 28 mV at the highest temperature of 195 °C. However, in all test cases, the repassivation ability of the age hardened alloy deteriorates. With the help of further analysis of the electrical potential, ΔE_2_, we can conclude that the most stable and corrosion resistant surface film is obtained in the quenched state of the alloy. There is also the biggest difference in electrical potential between hardened and unhardened specimens, which is 44 mV and is in favour of the hardened specimen. All other ageing temperatures show that the difference (Δ) between the hardened and unhardened alloy is small in all cases, and the surface film is always more stable in unhardened specimens. The expected precipitated MgZn_2_ phase (M-phase) represents cathode sites with a more positive electrochemical potential with respect to aluminium matrices, α-Al, so that anodic dissolution of the matrix occurs at the crystal boundaries of the precipitates. The dissolution of the aluminium matrix consequently affects the loss of individual separated phases, which explains the formation and development of corrosion damage. 

**Figure 8:**
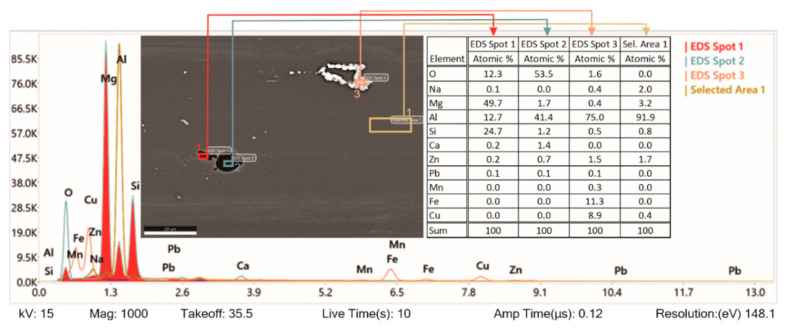
SEM image and EDS analysis of tested aluminium alloy.

Figure 8 shows further analysis of a quenched and shot peened specimen by EDS. The study shows that specimen preparation—grinding imprints small particles of SiO_2_, which are strongly bonded with the 7075-T6 matrix (EDS spot 1). When they fall off (EDS spot 2), they pull off the matrix around it too. Analysis of EDS Spot 3 shows that the chemical composition matches the precipitation of Al_7_Cu_2_Fe. As mentioned before, AA7075 is an Al–Mg–Zn–Cu alloy material normally comprised of the elements Al, Zn, Cu, Mg, Fe, and Cr (as shown in Table 1). However, in the EDS spectrogram, Si, O, and Ca can also be observed. These foreign materials typically appear as SiO_2_ or Al_2_O_3_, and they come as the remnants of the grinder used for the specimen preparation. SiO_2_ is chemically stable. It is an acidic oxide, which does not react with general acids or water.

**Figure 9:**
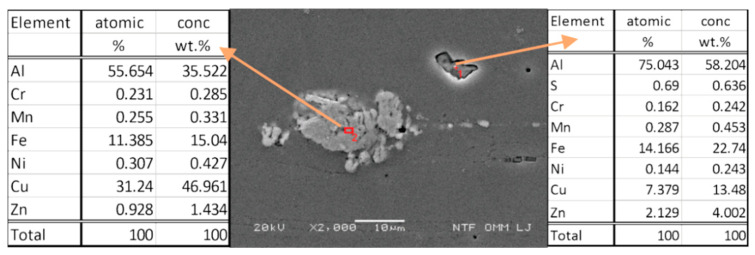
SEM image and chemical analysis of precipitates.

Figure 9 shows the SEM image just below the surface with an analysis of two precipitates. Intermetallic phases formed by solution heat treatment are based on the main alloying elements (Zn, Cu, Mg). When the separated phases are concentrated at the boundaries of the crystal grains, they cause intergranular corrosion and increase sensitivity to crack formation and growth due to stress corrosion. Since Cu (31.24%) is also present, we assume that the separated phases come close to the intermetallic compound Al_2_Cu in their chemical composition. The occurring intermetallic phases are present in the form of MgZn_2_, Al_2_CuMg, Al_6_Mg_11_Zn_11_, Al_7_Cu_2_Fe, Al_2_Mg_3_Zn_3_, Al_2_Cu, and Al_13_Fe_4_. Similar results were also presented by Fan et al. in their study [46]. 

**Figure 10:**
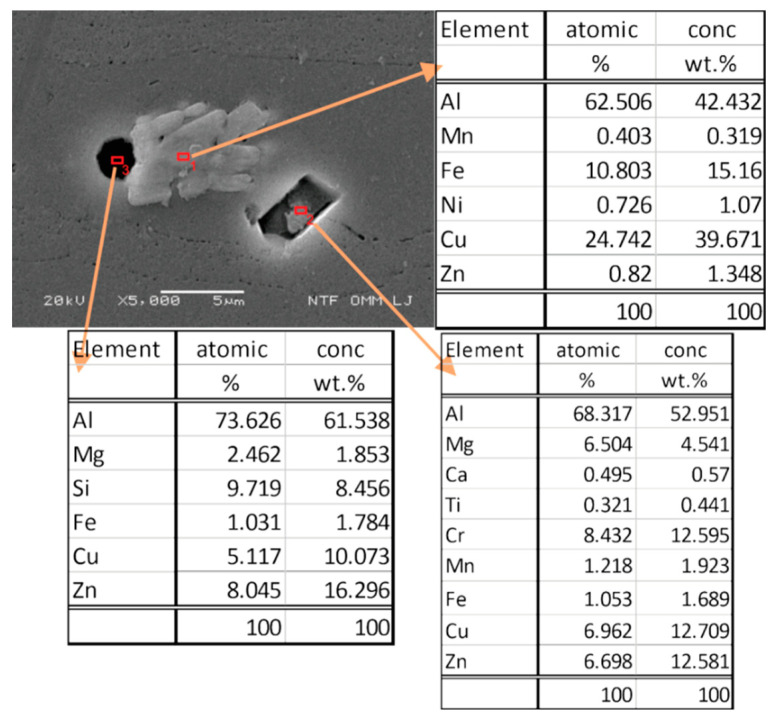
SEM image and chemical analysis of precipitates.

In Figure 10, the separate phases formed after solidification by solution heat treatment were microchemically analysed, and their chemical composition was found to be very close to the intermetallic phase of Al_7_Cu_2_Fe. All intermetallic phases in aluminium alloys with iron content are generally classified as impurities. Usually, these excluded phases have rather irregular shapes. Precipitates in the form of Mg_2_Si represent sites where passivation of the material is difficult, and they are localised sites for corrosion attack. Based on the analysed sites, we can say that these are also present in our specimens. Due to the high density of corrosion currents on the contaminated surface of specimens hardened with steel balls, a heavily corroded surface layer is observed. We can also see that, depending on the chemical composition of the other precipitate, it is most likely that precipitates are in the form of Al_13_Fe_4_ or Al_7_Cu_2_Fe. These precipitates represent the cathode in the anode matrix of the aluminium alloy and, as a result, the corrosion resistance of the material deteriorates. Depending on the exact alloy compositions, some of these phases may be insoluble. Of note is that Mg_2_Si is virtually insoluble in Al 7075 [47].

SEM images confirm the dissolution of the aluminium matrix by the action of galvanic cells near the separated iron-rich phases of the Al_x_ form (Fe, Mn). The aluminium matrix dissolves because it has a negative potential that acts as an anode compared to iron-rich secretions that act as cathodes in these cases. From the size of precipitation, we can conclude that the size of precipitation does not affect the deterioration of the corrosion properties of aluminium alloy, which is also reported by other authors in their research [48].

## 4. Conclusions

The experimental results provided information regarding the effect of corrosion behaviour on shot peening and heat treatment of the alloy. The key aspects of the study are:

(a). X-ray diffraction analysis of the surface shows the greatest difference in types of precipitates between the specimens in the quenched state and the specimens aged at 195 °C. Detailed analysis of the specimens shows that Al_2_CuMg precipitates dissolve in the specimen aged at 195 °C. If we connect this with corrosion resistance (Figure 7), we see that the specimen (AA195 + SP) has the highest corrosion current (i_corr_ = 0.0117 μAcm^−2^), which also indicates the highest susceptibility to corrosion and the fastest degradation per year (C.R. = 127.4 μm/Y).
(b). DSC analysis shows that solidification of the examined aluminium alloy begins with the growth of primary α-Al mixed crystals, followed by the crystallisation of the remaining melt into the intermetallic phase Al_13_Fe_4_, and finally, this is followed by the crystallisation of the phase η-MgZn_2_ phase. Again, most precipitates were found in the specimen (AA195 + SP) and because of that, corrosion resistance is the worst there as well. The number of precipitation phases is 5 times higher than in other specimens. The amount of precipitated phase indicates that the specimen was overaged.
(c). After shot peening we achieve maximum hardness of the surface layer, when artificial ageing of aluminium alloy at 145 °C is selected. However, the corrosion resistance analysis shows that the minimum values of corrosion current and corrosion rate is obtained at a higher ageing temperature of 170 °C.
(d). Cyclic polarisation curves of shot peened aluminium alloy with an Almen intensity 4A and 100% coverage shows that the most favourable results were obtained in the quenched and aged (at 170 °C) specimen. The volume share of precipitation phases is also about the same in these specimens, about 3.00%.
(e). EDS analysis of the specimen surface shows higher contamination with chemical elements that are not present in our aluminium alloy. They were imprinted during the grinding process.

## Figures and Tables

**Table 1:** Chemical composition of the selected aluminium alloy (wt.%).

Designation	Mg	Mn	Fe	Si	Cu	Zn	Cr	Ti	Zr + Ti	Al
AA7075	2.36	0.05	0.17	0.12	1.58	5.70	0.19	0.03	<0.01	bal.

**Table 2:** Roughness and microhardness parameters of treated AA7075.

Alloy State	HV_0.2_ before SP	HV_0.2_ after SP	R_a_ after SP
QS	155	157	1.91
AA145	175	185	2.02
AA170	160	175	2.19
AA195	105	118	2.53

**Table 3:** Reference temperatures of the onset of decomposition of the age-hardened phases.

Alloy State	Exothermic Precipitation Peaks [°C]				Endothermic Dissolution Peaks [°C]		
	1st	2nd	3rd	4th	1st	2nd	3rd
QS	109.6	220	243	/	148.4	/	/
AA145	110.6	182	247	296	147.6	216	265
AA170	119.1	186	304	/	149.8	232	/
AA195	110.4	210	/	/	149.3	258	/

## Data Availability

Not applicable.
